# Small intestinal transit in patients with liver cirrhosis and portal hypertension: a descriptive study

**DOI:** 10.1186/1471-230X-12-176

**Published:** 2012-12-08

**Authors:** Stine Karlsen, Lotte Fynne, Henning Grønbæk, Klaus Krogh

**Affiliations:** 1 Department Hepatology and Gastroenterology, Noerrebrogade 44, Aarhus University Hospital, DK-8000, Aarhus C, Denmark

**Keywords:** Liver cirrhosis, Portal hypertension, Gastrointestinal motility, Small intestinal transit time, Colonic transit time

## Abstract

**Background:**

Gastrointestinal dysmotility may be involved in the development of bacterial translocation and infection in patients with liver cirrhosis. The aim of the present study was to describe gastric, small intestinal and colorectal motility and transit in patients with liver cirrhosis and portal hypertension using a magnet-based Motility Tracking System (MTS-1) and standard radiopaque markers.

**Methods:**

We included 15 patients with liver cirrhosis (8 Child-Pugh A, 6 Child-Pugh B, and 1 Child-Pugh C) and portal hypertension (11 males, median age 54 years (range 38–73), median hepatic venous pressure gradient 18 mmHg (range 12–37)), and 18 healthy controls (8 males, median age 58 years (range 34–64)). The gastric emptying time and small intestinal motility were evaluated by MTS-1, and the total gastrointestinal transit time was assessed by radiopaque markers and abdominal radiographs.

**Results:**

The velocity through the proximal small intestine was significantly higher in cirrhotic patients (median 1.27 metres (m)/hour, range 0.82–2.68) than in the healthy controls (median 1.00 m/hour, range 0.46–1.88) (p = 0.03). Likewise, the magnet travelled significantly longer in both fast (p = 0.04) and slow movements (p = 0.05) in the patient group. There was no significant difference in either gastric emptying time—23 minutes (range 5–131) in patients and 29 minutes (range 10.5–182) in healthy controls (p = 0.43)—or total gastrointestinal transit time—1.6 days (range 0.5–2.9) in patients and 2.0 days (range 1.0–3.9) in healthy controls (p = 0.33). No correlation was observed between the hepatic venous pressure gradient and the velocity of the magnet through the small intestine.

**Conclusion:**

Patients with liver cirrhosis and portal hypertension demonstrated faster-than-normal transit through the proximal small intestine. This may be due to an overactive bowel, as suggested by previous studies.

## Background

Liver cirrhosis is a condition associated with increased morbidity and mortality. Clinically significant portal hypertension (>10 mmHg) may lead to decompensation, with complications such as ascites, oesophageal varices and hepatic encephalopathy (HE). Spontaneous bacterial peritonitis (SBP) can further complicate ascites with abdominal and systemic inflammation and lead to potentially fatal complications, such as variceal bleeding and HE [1].

Sanchez et al. proposed that gut bacteria may be translocated into the peritoneal cavity, contributing to the development of SBP [2]. An altered gastro-intestinal transit time (GITT) may enhance the intestinal bacterial overgrowth, subsequently increasing peritoneal bacterial translocation [3]. Therefore, small intestinal dysmotility may be involved in the development of cirrhotic complications.

Previous studies have suggested that cirrhotic patients demonstrate a prolonged gastric emptying time and decreased gastric compliance [4,5]. Other studies have demonstrated a prolonged total GITT related to complications of cirrhosis, such as spontaneous bacterial peritonitis and malnutrition [5,6]. Due to the difficulty of accessing the small intestine, the above studies primarily investigated gastric emptying and colonic transit time (CTT). The magnet-based Motility Tracking system (MTS-1) used in this study allows for a minimally invasive description of all segments of the gastrointestinal canal, including the small intestine [7].

We aimed to examine the small intestinal motility and transit times of the stomach, small intestine and colorectum separately. We hypothesised that small bowel motility was reduced and that segmental gastrointestinal transit times were prolonged in patients with liver cirrhosis.

## Methods

### Subjects

We included patients aged 18–75 years with liver cirrhosis of any origin referred for clinical investigation of the hepatic venous pressure gradient (HVPG) at the Department of Medicine V (Hepatology and Gastroenterology), Aarhus University Hospital, Denmark. If the HVPG was greater than 12 mmHg, the patient was invited to participate in the study. Of 62 consecutive patients, we included 15 (11 men, median age of 54 years, range between 38 and 73 years). In total, 38 were excluded because of comorbidities or the use of medications affecting bowel function; 6 patients did not wish to participate, and 3 patients were not included for practical reasons. Patients were compared to 18 healthy controls (8 men, median age of 58 years, range between 34 and 64 years). All patients and healthy volunteers included were without other known conditions affecting bowel function, and none had undergone abdominal surgery. All patient medications with known effects on gastrointestinal motility were ceased one day prior to beginning the investigation. All patients were characterised by their HVPG, Child-Pugh scores, and number of bowel movements per day.

The study was performed in accordance with the Declaration of Helsinki and was approved by the local ethics committee (reference number M-20110006). All subjects gave written informed consent before participation.

### Hepatic venous pressure gradient measurement

The portal pressure was measured indirectly by HVPG, as previously described [8]. Under radiographic guidance, an intravascular pressure catheter was placed in a hepatic vein through the femoral vein. The free and the wedged pressures were obtained, and the difference between them represents the pressure gradient across the liver [8].

### Gastric emptying and small intestinal motility

Gastric emptying, small intestinal transit time, median velocity of the magnet through the small intestine and contraction patterns were determined using the magnet-based Motility Tracking System-1 (MTS-1) (Motilis, Lausanne, Switzerland) [7]. The MTS-1 system provided information about gastrointestinal transit times and motility patterns by detecting the position of an orally ingested magnetic pill. Subjects ingested a silicon-covered magnetic pill (6×15 mm, density 1.8 g cm^−3^). A detection plate with an array of 4×4 sensors was placed in front of the subject and gave the position and orientation of the pill defined by three coordinates (position: x, y, z) and two angles (θ, φ). A 2D assessment was made on the magnet position within the subject by entering the detection plate location in relation to anatomical reference points, thereby defining the pill’s propagating movements. Two external sensors were placed on the subject’s neck and thorax, recording respiratory and movement artefacts.

The magnetic coordinate data were continuously sent to a computer at a sampling rate of 10 Hz during the investigation period. Custom-made software was used to picture the coordinates and angles as waves of different frequencies and amplitudes in real-time. The waves depicted by the data from the angles represented the gastro-intestinal contractions. By assessing this wave pattern, we could determine three different gastro-intestinal phases: the gastric phase, with a pattern of three contractions min^−1^ and medium amplitude; the small intestinal phase, with 10–14 contractions min^−1^[9] and high and mixed amplitude; and the colonic phase, with up to 6 contractions min^−1^ and low amplitude [10]. The movements through the small intestine were divided into the following three types: fast (>15 cm min^−1^), slow (between 1.5 and 15 cm min^−1^) and very slow (<1.5 cm min^−1^) [10].

The system calibration for ambient magnetic fields was performed before each recording and subsequently every 90 minutes. The technical specifications of MTS-1 and data on its validity have recently been published [7,10].

### Experimental protocol for the Motility Tracking System

After eight hours of fasting, investigations with MTS-1 started at 8 A.M. The outer abdominal size was estimated using the distances between the xiphoid process and the symphysis pubis, the superior iliac spines on both sides, and the abdominal wall and the back. The pill was ingested with 100 ml of water. During the measurements, the subject was placed on a wooden bed with the headboard in an upright position. Measurements were continued for a minimum of seven hours or until the magnetic pill was in the caecum. Small breaks for toilet visits and short walks were permitted at the subjects’ request. The subjects were encouraged to not talk, sleep or move but were allowed to read or watch TV. After the magnetic pill had passed to the duodenum, a standardised breakfast (1.991 kJ, 14% protein, 45% fat, 41% carbohydrate) was served. Three hours after breakfast, a standardised lunch (1.500 kJ, 16% protein, 32% fat, 52% carbohydrate) was provided. When the magnetic pill had passed to the caecum, or after a minimum of seven hours, the investigation was terminated.

### Total gastrointestinal transit time (GITT)

GITT was determined as described by Abrahamsson et al. [11]. At 12 A.M. for six consecutive days, the patient ingested a capsule containing 10 radiopaque markers. On the seventh day, a plain radiography of the abdomen was taken. The number of markers left in the different segments of the colon was counted, and the GITT was calculated based on the following formula:

$$\mathrm{GITT}=\left(M+\left(f\times D\right)\right)D$$

where *M* is the total number of markers left; *D* is the number of markers ingested each day; and *f* is the fraction of the daily markers selected for the provision of transit. In this case, *f* = 0.5.

### Data analysis

**Figure 1:**
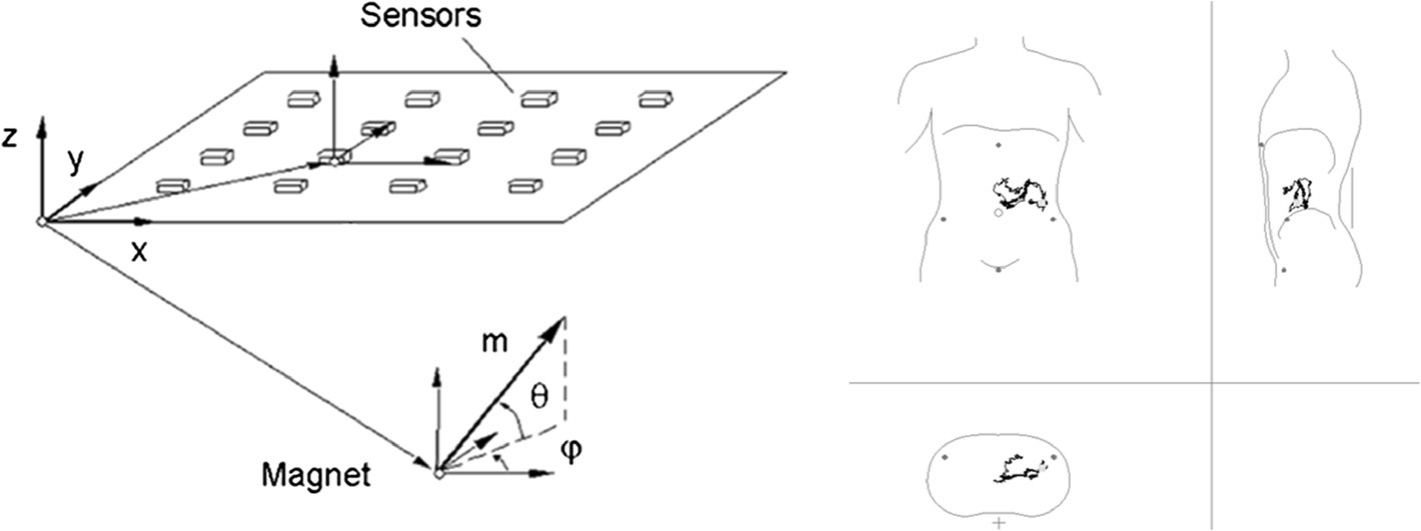
**Magnetic sensor and duodenal arch.** Tracking of the magnetic pill by 16 (4x4) external sensors. The position is defined by coordinates X, Y and Z, and the rotation is defined by the angles φ and θ (left). 3D representation pyloric and duodenal passage (right). The sequence displayed is 10 seconds long

Two investigators independently evaluated data obtained by MTS-1, and the mean values were used for all subsequent analysis. GE was the time from magnet ingestion to pyloric passage, identified by the following signs: the cessation of the characteristic gastric pattern (3 contractions min^−1^), the appearance of small intestinal contraction pattern (10–14 contractions min^−1^) and the appearance of the duodenal arch on the 2D representation (Figure 1). The small intestinal transit time was the time from pyloric passage to ileocaecal passage, identified by the cessation of contraction patterns of 10–14 min^−1^ and the occurrence of a short, fast movement of the magnetic pill situated in the lower right quadrant on the 2D representation. The mean velocity of the magnetic pill within the small intestine was determined using the MTS-1 software *MTS Tools* (Motilis, Lausanne, Switzerland) [7].

### Statistical analysis

All data are expressed as medians with ranges. GE, small intestinal propagation velocity and GITT were compared in the two groups using the Mann Whitney *U*-test. The correlations between HVPG and the presence of oesophageal varices and GI transit times were analysed using Spearman’s rank correlation. P < 0.05 was considered statistically significant.

## Results

Overall, 15 patients with cirrhosis and portal hypertension completed the study and were compared to 18 healthy controls. The aetiologies of cirrhosis were alcohol (n = 11), non-alcoholic steatohepatitis (n = 2), viral hepatitis C (n = 1) and primary biliary cirrhosis (n = 1). The Child-Pugh classifications were A (n = 8), B (n = 6) and C (n = 1). The median portal pressure was 18 mmHg (range: 12–37). Additionally, 10 out of 15 patients (67%) had previously bled from varices; 12 patients had grade 2–3 oesophageal varices; one patient had grade 1 oesophageal varices; and one patient had gastric varices without signs of oesophageal varices. One patient had no evidence of either oesophageal or gastric varices. Two patients with alcohol cirrhosis had current alcohol intake.

In all patients and healthy subjects, the number of bowel movements per day and the stool consistency were within normal limits.

### Gastric and intestinal motility

The MTS-1 procedure was well tolerated by all subjects. The characteristic basic contraction frequencies of 3 min^−1^ in the stomach and 9–11 min^−1^ in the small intestine were observed in all patients and controls. In both groups, the velocity of the magnetic pill declined as it passed distally through the small intestine. Data on gastric emptying and small intestinal motility are shown in Table 1. There was no significant difference in the median fasting state gastric emptying time between patients and healthy controls (p = 0.43). the magnet propagation velocity during the first two hours in the small intestine was significantly faster in patients than in healthy controls (p = 0.03).

**Table 1:** Gastrointestinal motility and transit times in patients with liver cirrhosis and in healthy controls

	**Patients with cirrhosis**	**Healthy controls**	**p**
Gastric emptying time (minutes)	23 (5–131)	29 (11–182)	0.43
Magnet velocity during first 2 hours in the small intestine (meters/hour)	1.27 (0.82–2.68)	1.00 (0.46–1.88)	0.03
Magnet velocity during first 4 hours in the small intestine (meters/hour)	0.95 (0.60–1.79)	0.75 (0.30–1.34)	0.06
Total gastrointestinal transit time (days)	1.6 (0.5–2.9)	2.0 (1.0–3.9)	0.33
Duration of time in the small intestine with *fast movements* (minutes)	8 (3–18)	6 (2–14)	0.16
Distance travelled during *fast movements* during the first 4 hours (meters)	1.97 (1.28–4.98)	1.47 (0.27–3.23)	0.04
Duration of time in the small intestine with *slow movements* (minutes)	50 (19–87)	38 (10–76)	0.45
Distance travelled during *slow movements* during the first 4 hours (meters)	1.27 (0.58–2.07)	0.89 (0.30–1.66)	0.05
Basic frequency of small intestinal contractions (contractions/minute)	9.5 (8.8–10.9)	9.8 (8.7–10.4)	0.18

The magnet pill moved faster through the upper small intestine in patients than in healthy controls. All data expressed as median (range).

All participants stated that they had followed the protocol for the assessment of GITT. The median GITT was 1.6 days (range 0.5–2.9) in patients and 2.0 days (range 1.0–3.9) in healthy controls (p = 0.33). GITT was more than 3 days in one healthy subject (3.4 days), while none of the patients had a GITT longer than 3 days.

### Portal hypertension and small intestinal motility

We found no correlation between the degree of portal hypertension and the velocity of the magnetic pill through the small intestine (r = −0.29, p = 0.29). There was no difference in the propagation velocity during the first two hours between the patients with no or grade 1 oesophageal varices and those with grade 2–3 oesophageal or gastric varices (p = 0.93).

## Discussion

In the present study, we evaluated the small intestinal motility and transit in patients with liver cirrhosis and clinically significant portal hypertension. We used a novel magnet-based MTS-1 that provides detailed information on intestinal motility. Our main finding was that patients with cirrhosis and portal hypertension had faster-than-normal postprandial transit through the proximal small intestine. Thus, our hypothesis of reduced small bowel motility and prolonged segmental gastrointestinal transit times was rejected.

The basic and highly characteristic contraction frequencies of the stomach and small intestine were unaltered by portal hypertension, but the distance was covered faster both during the long periods with relatively slow transit and during the short bursts of very fast movement. Madsen et al. studied eight patients with cirrhosis and portal hypertension using scintigraphy [12]. They found no difference in the GE of solids or small intestinal transit times compared to healthy controls. The scintigraphy technique is considered the gold standard for measuring GI transit times. However, standard 30 minute-intervals between each image were used, and smaller differences in either GE or small intestinal transit times could thus have been missed. In the same study, cirrhosis patients showed faster-than-normal colonic transit times [12]. It has previously been demonstrated that patients with cirrhosis have a prolonged phase II of the fasting small intestinal migrating motor complex (MMC) [13]. We obtained data on small intestinal motility in the postprandial state, but our data also suggest an abnormally active small intestine in patients with liver cirrhosis.

Some previous studies have suggested that patients with liver cirrhosis have prolonged GE [14,15], while others found no difference [16]. The main focus of the present study was small intestinal motility, aiming for an exact determination of pyloric passage that MTS-1 provides [10]. As a detailed evaluation of GE should be performed in both the solid and the liquid phases, MTS-1 is not ideal for the description of GE. Large particles will typically pass through the pylorus during phase III of the migrating motor complex [17]. Unless magnet intake is standardised with respect to MMC or with a meal, a large variation in solid-state GE should be expected, as observed in the present study.

Our findings are in contrast with some previous studies that demonstrated prolonged gastrointestinal transit times in patients with liver cirrhosis. However, those studies investigated the total orocaecal transit times with radiopaque markers [6] or lactulose breath tests [18]. These methods do not differentiate between GE and small intestinal transit time, and differences in small intestinal transit may not be detected, especially if GE is prolonged. Furthermore, patients with liver cirrhosis often suffer from small intestinal bacterial overgrowth, making the lactulose breath test unreliable [19]. Other studies have shown both prolonged and reduced colonic transit times in cirrhotic patients [6,20]. However, GITT mainly reflects colonic transit time, and in our study, there was no difference in GITT between patients with cirrhosis and controls.

It has previously been shown that a history of SBP correlates to the degree of GI alterations in patients with liver cirrhosis [21]. Additionally, there are indications that abnormalities in small intestinal motility are related to the degree of chronic liver failure [5]. In accordance with the present study, Madsen et al. found no correlation between transit times and HVPG [12]. However, this may be a type II error due to the small number of patients included in both studies.

Our findings were surprising and contrary to our hypothesis. It is unclear through which mechanisms portal hypertension may affect gastrointestinal motility. It may be that portal vein and intestinal wall blood stasis may cause an overactive bowel. We studied small intestinal motility in the postprandial state. In the fasting state, a marked change in the contraction patterns of phase II and the MMC has been previously observed [13]. Whether this behaviour indicates an overactive bowel is unknown. In healthy individuals, intestinal peristalsis, gastric acid and mucosal immunity act to protect the small intestine from bacterial overgrowth and translocation. Breath tests for small intestine bacterial overgrowth are not reliable for research purposes [22], and cultures from small intestinal fluid require invasive investigation. For these reasons, we did not investigate whether patients in the present study had small intestine bacterial overgrowth.

Previous studies have suggested that the aetiology of liver cirrhosis may influence GI transit times [3,23]. In the present study, we included patients consecutively and independently of cirrhosis aetiology. The mixed aetiology and differing representation of Child-Pugh classes in a small sample size are major limitations of the study. The main aetiology of cirrhosis was alcohol consumption, and it is well known that alcohol may affect gastrointestinal motility. However, all but two patients had abstained from drinking for at least 3 months prior to the investigation. Pancreatic insufficiency may also reduce small intestinal transit. However, none of the patients had a history of acute or chronic pancreatitis or malabsorption, and no signs of calcification in the pancreatic area were noticed on abdominal radiographs. Furthermore, autonomic dysfunction in patients with liver cirrhosis may lead to gastroparesis and an abnormal frequency of gastric contractions [24]. Compared to healthy controls, we found no differences in the contraction frequency in either the stomach or the small intestine.

There was a difference in gender between our patients and the control group. However, when stratifying the data in each group by gender, we could not detect any differences in GE, small intestinal velocity or colonic transit time. Ten out of 15 patients had a history of bleeding oesophagus varices. Oesophago-gastro-doudenoscopy had been performed in all patients, and 14 had oesophageal varices. We found no association between the presence of varices and motility patterns.

## Conclusion

In conclusion, our findings indicate that GI transit is abnormally fast in the proximal small intestine of patients with liver cirrhosis and portal hypertension. More and larger studies are needed to confirm our findings and to determine the mechanisms of action. Furthermore, the associations among gastrointestinal dysmotility, bacterial overgrowth and the risk of cirrhotic complications should be studied.

## Competing interests

The authors declare that they have no competing interests.

## Authors’ contributions

SK: Involved in study design, inclusion of patients, all study related procedures, data analysis and writing first draft of the manuscript. LF: Involved in study design, study related procedures, data analysis and interpretation. HG and KK: Initiated the study, involved in study design, inclusion of patients, study procedures, data analysis and interpretation. All authors have participated in writing and finalising the manuscript, and all have approved the final submitted version of the manuscript.

## Pre-publication history

The pre-publication history for this paper can be accessed here:

http://www.biomedcentral.com/1471-230X/12/176/prepub
